# Adolescent experience of radically open dialectical behaviour therapy: a qualitative study

**DOI:** 10.1186/s12888-022-04114-8

**Published:** 2022-07-14

**Authors:** Julian Baudinet, Charlotte Watson, Phillipa Louise Brothwood, Rhian Parham, Lindsay Smith, Natasha Snowden, Anna Konstantellou, Katrina Hunt, Mima Simic

**Affiliations:** 1 Maudsley Centre for Child and Adolescent Eating Disorders (MCCAED), Maudsley Hospital, De Crespigny Park, Denmark Hill, London, SE5 8AZ UK; 2 Institute of Psychiatry, Psychology & Neuroscience, King’s College London, De Crespigny Park, Denmark Hill, London, SE5 8AZ UK; 3 South London and Maudsley NHS Foundation Trust, London, UK

**Keywords:** Adolescent, Depression, Eating disorders, Self-harm, Radically open dialectical behaviour therapy, Dialectical behaviour therapy, Overcontrol

## Abstract

**Background:**

Radically Open Dialectical Behaviour Therapy (RO DBT) is a novel transdiagnostic treatment that targets ‘maladaptive overcontrol’; a transdiagnostic cluster of traits associated with excessive emotional and behavioural inhibitory control. Outcomes are promising for adults with a range of psychiatric disorders. No study to date has explored the adolescent experience of RO DBT.

**Methods:**

Of the 25 eligible adolescents who received RO DBT between March 2015 and April 2017, 15 (14–17 years) consented and completed a semi-structured interview about their experience of treatment within 1 month of discharge. Interviews were recorded and then transcribed manually. Free text responses were analysed using reflexive thematic analysis.

**Results:**

The majority (*n* = 13) had a primary diagnosis of anorexia nervosa, although comorbidity was the norm, with 80.0% having two or more predicted comorbid psychiatric diagnoses. All had received some prior psychological treatment. Four themes were identified from analysis of transcripts: 1) Broadening Horizons, 2) Building Connections, 3) Flexibility, 4) Information Overload. Generally, RO DBT was perceived as helpful in both content and process. The focus on social and broader well-being, rather than specific mental health symptoms, was considered beneficial by many. Adolescents appreciated the group-based format of skills classes and reported benefiting from learning and practicing skills each week. The fourth theme, Information Overload, highlighted that for some, the amount of content felt overwhelming and that it was hard to remember and digest all the information, suggesting that adaptations, or simplifications, may be required to ensure accessibility for adolescents.

**Conclusions:**

RO DBT is perceived as a relevant and beneficial new treatment for adolescents with maladaptive overcontrol. The broad treatment focus is perceived as unique and of particular benefit. It is reported to help with general and social functioning and foster cognitive and behavioural flexibility. Nevertheless, the amount and complexity of material was felt to be very large by some and may suggest the need for modified adolescent-specific materials.

**Supplementary Information:**

The online version contains supplementary material available at 10.1186/s12888-022-04114-8.

## Background

Radically Open Dialectical Behaviour Therapy (RO DBT) is a relatively new treatment that targets maladaptive overcontrol, a transdiagnostic cluster of characteristics associated with excessive inhibitory control [1, 2]. This cluster includes several, interrelated characteristics such as supressed emotional expression, cognitive and behavioural inflexibility, increased threat sensitivity and reduced reward processing [1, 3]. These are often associated with increased social isolation and reduced social connectedness [1, 4, 5], as well as psychiatric diagnoses of refractory depression [1, 5], restrictive eating disorders [4, 6, 7], obsessive-compulsive personality disorder [1], and paediatric anxiety disorders [3].

Overcontrol can be expressed discreetly, and difficulties are not always overtly obvious when interacting with others. Neurobiological, environmental and learning factors are all hypothesised to contribute to overcontrol [1]. Given the high rates of comorbidity [8–10], relapse [11–14], and treatment non-response [10, 15, 16] for individuals with the aforementioned cluster of psychiatric diagnoses, treatments that target underlying transdiagnostic mechanisms and reconceptualise treatment targets to the management of broader temperamental and personality factors may help to improve outcomes and reduce relapse rates.

In a recent randomised controlled trial (*N* = 250) for adults with refractory depression, RO DBT was associated with a greater reduction in depressive symptoms compared to treatment as usual at the end of treatment (7 months), but not at follow-up [5]. Other preliminary evidence suggests it is helpful at supporting symptom improvement for adults with anorexia nervosa [17, 18], as well as global distress and general well-being for adults with autism spectrum disorders [19]. Only one study to date has investigated the experience of RO DBT [20], which was with a sample of adults with anorexia nervosa who experienced the treatment as beneficial and acceptable.

Adaptations of RO DBT have recently been described and evaluated using uncontrolled case series designs in both the outpatient [21] and day patient setting [7] for adolescents with eating disorders, anxiety and/or mood difficulties. In both treatment contexts improvements are observed in psychiatric symptoms (eating disorder and mood), relationship quality, social withdrawal, cognitive flexibility, reward sensitivity and the maladaptive use of emotional suppression to regulate emotions [7, 21]. These adaptations were based on informal feedback from adolescents that some of the RO DBT materials were more relevant for adults. Adaptations in these uncontrolled case series’ include shortening treatment length (20 skills classes in the outpatient setting; 16 in a day patient setting instead of 30 skills classes), shorter classes (60–90 min vs 2.5 h) and amending materials to include more adolescent-focused examples and teaching materials [7, 21]. This fits with the current study, in which RO DBT was very slightly amended (see method section).

Given the emerging nature of RO DBT in the literature, especially for adolescents, qualitative studies are needed to understand how the treatment is relevant and whether any further adaptations are needed to tailor the treatment for this age group. This study aimed to begin to address this by exploring the experience of RO DBT treatment for adolescents with eating disorders, depression and/or anxiety. The aim is that this will then lead to further refinement of the RO DBT model for the adolescent population, specifically.

## Method

### Sample

A convenience sample of all adolescents who engaged in RO DBT between March 2015 and April 2017 were eligible for this study. Those who engaged in 4 out of 30 skills classes or fewer were considered non-starters and were not eligible given their experience of RO DBT was so limited.

All adolescents were referred by a mental health clinician (psychologist, psychiatrist, family therapist or mental health nurse) from either the National and Specialist Dialectical Behaviour Therapy (DBT) service or the Maudsley Centre for Child and Adolescent Eating Disorder Service (MCCAED) for treatment of transdiagnostic difficulties associated with overcontrol. Both are public, specialist, services for children and adolescents in England.

All adolescents referred from the DBT service were assessed for over- and undercontrol as part of their initial assessment. Adolescents identified as more overcontrolled were then referred to RO DBT, as opposed to standard DBT. Those referred from MCCAED had all engaged in some type of eating disorder treatment within MCCAED, typically family therapy, and were referred for RO DBT after minimal or partial treatment response. This was determined by the mental health clinician offering the eating disorder treatment with the support of their supervisor and multidisciplinary team in case-discussion meetings. Partial treatment response was defined as ongoing difficulties with distress around mealtimes and/or high levels of eating disorder behaviours and/or cognitions that interfered in daily functioning. All had achieved partial or full weight restoration by the time of referral to RO DBT. See below for details on the screening process for overcontrol.

### Treatment intervention and model

All adolescents in this study were offered manualised RO DBT [1, 2]. It consists of 30 weekly 90-min skills classes and a weekly 60-min individual session. Between two to eight adolescents attend skills class at any one time and work together with one or two facilitators depending on the group size. Parents do not attend skills classes. Facilitators were mental health clinicians from either the DBT service or MCCAED. All were qualified mental health clinicians with undergraduate and postgraduate level education and clinical training. All had attended 10-days of intensive RO DBT training and received regular supervision provided by an approved supervisor from the Radically Open Dialectical Behaviour Therapy Institute.

Skills classes aim to teach new skills to manage maladaptive overcontrol and include in-session mindfulness practice, homework provision and review. Skills taught focus on helping individuals to understand the neurobiological underpinnings of emotional overcontrol, to live more flexibly, more openly express their emotions, be open to feedback, and engage in novel behaviours. There is a strong focus on improving social connection and monitoring/adjusting social signalling in line with identified value-based goals.

Individual sessions support individuals to put these skills into practice. This includes the use of diary cards, in-session role plays, addressing alliance ruptures and the use of chain and solution analyses. See the treatment manual for further details of treatment aims and structure [1, 2].

Two minor treatment amendments were made to the original RO DBT material in this pilot. Firstly, skills classes were 90 min, as opposed to 2.5 h, in duration. Secondly, a one-off parent education group was offered. All parents with an adolescent who had commenced RO DBT were invited to attend. This one-off group session provided information on the treatment model, the concept of overcontrol and how it can influence mental health.

These two minor amendments were made to make the treatment fit with the adolescent population. A briefer class, held in the evenings (16.00–17.30), was decided on so that adolescents could attend without missing too much school. The parent psychoeducational group was included based on guidance that it is generally best practice to include caregivers in child and adolescent mental health treatments. The one-off group was designed to meet this need, whilst maintaining fidelity to the treatment model.

Any variations in treatment length were recorded in this pilot study group. An individual’s treatment length in this study was determined by consensus between the adolescent and RO DBT clinician/consult team. Early discharge (< 30 skills classes) was considered once the young person had met their therapeutic goals. Prolonged treatment (> 30 skills classes) was considered if the adolescent and team agreed more would be beneficial and the young person was using treatment effectively.

### Ethics approval and consent to participate

This study was approved by the SLaM CAMHS Service Evaluation and Audit Committee, which is the approving body for such projects within SLaM. To ensure all participants were fully aware and consenting to participation and publication of results, informed consent was also obtained, including parental informed consent for young people under 16 years of age.

### Screening and diagnostic assessment tools

All adolescents in this case series completed the Development and Wellbeing Assessment (DAWBA) [22] prior to attending either service, followed by a clinical interview at assessment. The DAWBA is a structured diagnostic tool that generates ICD-10 [23] and DSM-5 [24] predicted psychiatric diagnoses for two to seventeen-year olds. It has good reported validity as a diagnostic tool [25]. Primary diagnosis (eating disorder [MCCAED] or depression and/or anxiety [DBT service]) was confirmed during the clinical interview. A thorough assessment of comorbid diagnoses was not conducted. As such, predicted DAWBA comorbid diagnoses are presented separately to confirmed primary diagnoses.

The Assessing Styles of Coping Word-Pair (ASC-WP) checklist [1] was used as the initial screening instrument for overcontrol. The ASC-WP is a 47-item self-report measure that requires participants to choose a word that best describes them from a pair of words displayed side-by-side. Word pairs include one word that is more representative of overcontrol and the other of undercontrol. The ASC-WP was in the process of being validated with adolescents at the time of data collection. Data has since been published and the slightly adapted version renamed the Youth Over- and Under- Control screening measure (YOU-C) [26]. In the current study, this initial screen with the ASC-WP was followed by clinical interview assessing overcontrol factors such as risk aversion, perfectionism, emotional expressiveness, social connectedness, and rigid and rule governed behaviour. These assessment interviews were conducted by RO DBT clinicians delivering the treatment.

### Procedure

All eligible adolescents were approached by a member of the RO DBT clinician team upon discharge. One-off, face-to-face interviews were completed in the clinic with just the adolescent and interviewer present. Interviews were conducted by Assistant Psychologists. All were female, had some knowledge of RO DBT theory, but were not involved in treatment delivery. They had some prior relationship with participants, as the Assistant Psychologists were involved in collecting routine outcome measures. All were completed within 1 month of discharge from RO DBT. Interviews were audio-recorded and then written transcripts generated manually. No field notes were made. To ensure uniformity, an interview topic guide was given to all interviewers (see Supplementary material for further details).

### Analysis plan

The interviews were analysed using Reflexive Thematic Analysis [27] within a critical realist framework, which views meaning and experience as subjective and influenced by social and cultural context. An inductive and semantic approach was used to analyse the data. The two analysing authors (CW and PLB) were guided and directed by the explicit content of the data rather than making assumptions, making inferences or looking at existing concepts [28, 29]. RO DBT is a relatively new treatment, and it was therefore felt that this approach would be most informative.

The six phases of thematic analysis outlined by Braun and Clarke were followed [29]. The analysing authors independently reviewed the data, spending time to become familiar with, and immersing themselves, in the data. Codes and themes were generated independently prior to the analysing authors meeting to discuss findings. Through four one-hour meetings, with time in between to allow for further reflection, the themes were revisited and reviewed before a final consensus was reached on themes that best reflected the data. This consensus was reached with no difficulties as the themes identified individually were easily mapped onto each other. As noted by Braun and Clarke [27], theme generation is a ‘creative and active process’ and with this flexibility, the absence of a coding framework in reflexive analysis and the recursive and reflective nature of the analysis, theme frequency was not noted.

It is worth reflecting that the two analysing authors, PLB and CW, approached the thematic analysis from two very different viewpoints. CW has knowledge of, and experience delivering RO DBT, whereas PLB has had no prior experience with RO DBT. It was felt that this provided an extra depth to the thematic analysis and in the formation of themes as the different perspectives were discussed before a consensus of the final themes was reached.

## Results

### Sample characteristics

Fifteen adolescents (14–17 years old) who participated in RO DBT during the pilot period completed interviews for this study. Two (13.3%) were referred from the CAMHS DBT service and 13 (86.7%) from MCCAED.

During the study period a total of 31 adolescents were referred for RO DBT, 3 from the DBT service and 28 from MCCAED. Of these, 7 (22.6%) were early dropouts (< 4 skills classes; 0 classes = 3, 2 classes = 2, 3 classes = 1, 4 classes = 1) and were not approached to participate. Of the remaining 24 who engaged, 19 (79.2%) completed treatment and 5 (20.8%) self-discharged before reaching their treatment goals (later dropout). Fifteen (62.5%) agreed to participate and completed an interview. None of the four who dropped out of treatment completed an interview, although they were approached. There were no differences between those who agreed to complete an interview and those who did not regarding age at commencement of RO DBT (*t*(22) = .56, *p* = .58), treatment length in weeks (*t*(21) = .86, *p* = .40), or percentage of median Body Mass Index (%mBMI; *t*(15) = 1.01, *p* = .33). Those who did not complete an interview attended significantly fewer skills classes (*t*(22) = 2.85, *p* = .009) and individual sessions (*t*(21) = 2.72, *p* = .01). See Table 1 for demographic information.

**Table 1:** Baseline demographics

Mean age (sd, range)	
- At commencement of RO DBT	16 years (1.3, 14–17)
- At time of interview	17 years (1.2, 15–18)
Gender distribution (%)	13 female (86.7%)
	2 male (13.3%)
Ethnicity	
- White British	15 (100%)
DSM-V Primary Diagnosis	
- Eating disorder	13 (86.7%)
- Anorexia Nervosa	12 (80.0%)
- Atypical Anorexia Nervosa	1 (6.7%)
- Major Depressive Disorder	2 (13.3%)
DAWBA Predicted DSM-V Comorbid Diagnoses	
- Major Depressive Disorder	4 (26.7%)
- Anxiety disorder (> 1 diagnosed)	10 (66.7%)
- Generalised Anxiety Disorder (GAD)	9 (60.0%)
- Social Phobia	6 (40.0%)
- Specific Phobia	2 (13.3%)
- Obsessive Compulsive Disorder (OCD)	2 (13.3%)
Self-harm present	8 (53.3%)
Number of predicted DAWBA diagnoses	
- 1 diagnosis	3 (20.0%)
- 2 diagnoses	5 (33.3%)
- 3 diagnoses	5 (33.3%)
- 4 or more diagnoses	2 (13.3%)
Previous treatment	
- FT-ED	12 (80.0%)
- CBT	4 (26.7%)
- DBT	2 (13.3%)
- Inpatient treatment	4 (26.7%)
- Day programme treatment	3 (20.0%)
- No previous treatment	0 (0.0%)

*Abbreviations*: *CBT* Cognitive Behaviour Therapy, *DAWBA* Development and Wellbeing Assessment, *DBT* Dialectical Behaviour Therapy, *FT-ED* eating disorder focused family therapy, *sd* standard deviation

### Treatment characteristics

The mean duration of RO DBT treatment was 28.6 weeks (sd = 9.9, range = 15–44.5, median = 29). This included a mean of 19.3 skills classes attended (sd = 7.3, range = 8–34, median = 17) and 18.4 individual sessions (sd = 10.9, range = 9–42, median = 15). Overall attendance of skills classes was high. Approximately half (*n* = 7/15, 46.7%) the adolescents did not miss a single skills class and two-thirds missed two or fewer (*n* = 10/15, 66.7%).

Percentage of median Body Mass Index (%mBMI) was recorded for 11 people at assessment. It was broadly within the healthy range (Mean = 93.7%mBMI (sd = 6.2, range = 79.6–103.9) and did not significantly change over treatment (*p* < 0.05).

### Qualitative findings

Four themes were identified following analysis of the transcripts. These were 1) Broadening Horizons, 2) Building Connections, 3) Flexibility and 4) Information Overload. Subthemes were also identified (see Table 2). Each are described below with relevant illustrative quotations. These are written verbatim, with only minor adjustments to spelling or grammar to aid ease of reading.

**Table 2:** Themes and subthemes

Themes	Subthemes
Broadening Horizons	- Different perspectives - You are more than your diagnosis
Building Connections	- You are not alone - Self connection - Reaching out
Flexibility	- Treatment - Structure - Application
Information Overload	No subtheme

#### Broadening horizons

The adolescents noted that the experience of RO DBT helped them to expand their viewpoint.

##### Different perspectives

The adolescents spoke about how RO DBT had broadened their perspectives and way of viewing the world. RO DBT challenged their usual way of thinking, “helping me notice things differently”. They noted that there are ‘alternative possibilities to overcontrol’. It also widened their world view; ‘[Therapy] did actually challenge me and encourage me to do new things and to try new things’. They also found that they were able to think about things differently, ‘putting yourself in [different] situations [in which I’m more] vulnerable’. The element of group work also led to ‘different people’s opinions, [and this] shows you different people’s perspectives’.

##### You are more than your diagnosis

The adolescents liked that the RO DBT topics and skills were not diagnosis specific and ‘focused on other things’ with one adolescent commenting, therapy was about ‘how to change in general as a person not just to do with eating disorders’. This more generic approach meant that the learning from RO DBT was generalisable and “can apply to everything”. This meant they were able to learn skills that ‘I can use [with] friends at school and when [I] am in other groups’. They also noted that ‘in different areas and in different stages of life, you are going to come across one of the aspects of RO we talked about’. One adolescent also talked about getting their family involved: ‘[I] Had to teach my dad and he was saying how helpful he found it, [it] shows [it is] helpful for everyone’.

#### Building connections

##### You are not alone

The adolescents spoke about the ‘benefit of sharing in a group’ and hearing other people’s experiences. Having the opportunities to talk and socialise with others led to a sense of togetherness, which was helpful combined with the individual sessions which were an ‘opportunity to discuss more personal things’. One adolescent noted that ‘It’s different in that I was introduced to more people my age that were, like, going through similar things’. The support you can give to others from sharing was also noted ‘by inputting in a group you are helping other people as well.... and that encourages them to input into a group as well’. One adolescent noted that by working in a group you practiced skills naturally so that you were ‘learning on the go’. Another commented that it seemed less like therapy. More than one adolescent commented that the combination of group and individual work was helpful.

##### Self-connection

The adolescents reported a greater self-awareness after the programme, having built stronger connections with their ‘self’. Adolescents commented that it gave a ‘third person perspective on your life’ and that ‘it helped me learn...how I come across to people’. One even noted ‘since using it, [I] have been learning something new about myself practically every day so it’s really helpful’. They also connected more to their emotional states; a ‘gradual process of identifying what I was feeling’ and noted how their behaviour can affect interactions; ‘Body language, open and intimate, how being a certain way can affect your relationships - [I] look back and now change my behaviour’.

##### Reaching out

Adolescents spoke about how the treatment helped them build social connection with others. This was noted by one adolescent who commented, treatment ‘focused on relationships and how you interact socially and I have never been good at that, [it] definitely helped’. The adolescents spoke about feeling more confident and relaxed in social situations by learning how to ‘interact with people’ and ‘signal emotions to someone’. This led to ‘increased confidence by working in groups’.

#### Flexibility

Adolescents described flexibility in multiple aspects of RO DBT and its application.

##### Structure

It was noted that the atmosphere of the therapy was in a sense building on flexibility as one adolescent described it as ‘more relaxed’. This was also mirrored in the individual sessions where content was specifically tailed to each individual. This was perceived as helpful and relevant.

##### Treatment

Adolescents commented that the programme encouraged them to ‘let go of rigidity’ and ‘challenge[d] the idea that behaviour must be really strict and regulated in order to feel safe’ as ‘these safety behaviours would impede us in social life’ and that there were ‘alternative possibilities to overcontrol’. One adolescent noted that ‘spontaneous things...occur from doing new things and being able to deal with what happens has been really helpful’. Multiple adolescents discussed a sense of loosening control and that there was less rigidity in their communication with others. Many also noted that there was a sense of progression, ‘[it is] really important to reiterate that when you using RO it is difficult at first, ...but [the] more you use skills in RO, it becomes easier’.

##### Application

Another example of flexibility was that the adolescents could pick and choose from a ‘toolbox of skills’. Examples of skills mentioned were: mindfulness, big 3 + 1 and push backs/don’t hurt me. One adolescent commented that there were ‘skills you use immediately and also daily ones e.g. mindfulness [as well as] stuff that helped over [a] long[er] period of time’.

#### Information overload

Adolescents noted that the treatment was intense at times, with one describing ‘information overload’ and another commenting ‘it is just a lot and [I] realised quite early on I wasn’t going to remember everything’. A few also felt that there was ‘repetition’ and asked for ‘less theory please!’. Furthermore, some adolescents fed back that the structure of the sessions was not always clear and that they would have liked more clarity over skills. One adolescent suggested that ‘maybe a skills plan would be useful” so that things would be clearer. Adolescents also commented that jargon was a feature often used and some requested less homework. Another adolescent commented that there were ‘acronyms … a lot [of] acronyms’.

## Discussion

This study aimed to explore the adolescent experience of RO DBT, a new transdiagnostic treatment for overcontrol. Generally, adolescents in this group found the treatment unique and beneficial in both content and process, and attrition was within expected limits for child and adolescent studies [30], suggesting acceptability.

Four themes were identified in the analysis. The first, ‘broadening horizons’, highlighted that adolescents felt RO DBT helped them to expand their viewpoint. They valued being challenged to see things from different perspectives and that skills focused on social and general well-being, rather than on reducing psychiatric symptoms, were valued. The benefit of RO DBT being relationship focused was highlighted by the second theme, ‘building connections’. This was multifaceted, in that adolescents spoke about connecting more with others, as well as their own internal experiences as a result of treatment. In the third theme, ‘flexibility’, they described how RO DBT helped them to let go of rigidity and be more spontaneous. They noted that both the treatment structure and content helped enable this. The fourth theme, ‘information overload’, indicated that many felt there was a large amount of material to cover, which was not always adolescent focused and contained jargon. Several noted that there was no way that they were going to be able to remember all the material, despite having handouts available and the use of acronyms to aid memory.

These findings generally fit with the only other qualitative study that investigated the patient experience of RO DBT. In a sample of 13 adults with anorexia nervosa / atypical anorexia nervosa (mean age = 25, sd = 6.02, range = 18–41), Isaksson, et al. [20] reported that patients found the treatment to be comprehensive and useful. RO DBT was found to promote flexibility, social connection, building trust with others and within the therapeutic relationship, and moving towards value-based goals. The similarities in experience between the two groups is notable and suggests applicability of RO DBT across the lifespan for people with anorexia nervosa, specifically, as this was the primary diagnosis for the majority of the current sample.

The fourth theme, ‘information overload,’ was a point of difference between the current study and the adult sample described by Isaksson et al. [20]. This finding suggests that adolescents, and possibly others, may benefit from modified, simplified, abbreviated, or adapted RO DBT treatment and/or materials that is developmentally attuned.

Another point of difference between the current data and other studies investigating RO DBT was treatment length. It was relatively short in the current study (mean = 29 weeks, 19 skills classes) compared to the prescribed length of 30 skills classes in the RO DBT manual [2] or 27 described in the largest RO DBT randomised controlled trial [5]. As aforementioned, treatment length in this study was determined by consensus between the adolescent and RO DBT clinician/consult team. Only two people attended slightly more than 30 skills classes (one attended 31, another 34), with the remainder attending fewer than the full round.

This observed reduction could be interpreted in several ways. One interpretation could be that adolescents require a slightly smaller treatment length. More likely, however, is that shorter treatment was observed due to the sample’s prior treatment experiences. All adolescents in this study participated in at least one form of treatment prior to RO DBT, the majority being eating disorder focused family therapy. This prior therapeutic input within the same wider clinic/team would likely have reduced RO DBT treatment length.

## Limitations and future directions

Despite several strengths, there are some important limitations to this study. Methodologically, the analysis would have been strengthened by a third reviewer, who was not involved in the direct analysis of the data but who was included in reviewing the findings. Member checking with the participants would have also strengthened the analysis.

Most notable, however, is the relatively small and homogeneous sample, making the findings difficult to generalise. All adolescents were white British, nearly all were female and all were treatment completers. Furthermore, even though two services treating different diagnostic groups were referring adolescents to RO DBT in this study, the vast majority who were referred and participated came from MCCAED, a much larger service. This meant that nearly all had a primary diagnosis of anorexia nervosa, had some experience of eating disorder treatment and were mostly weight restored.

Future research is needed to replicate the current findings and extend them to more diverse groups, including treatment non-completers. It will be important to further test whether adolescents who have no treatment history, or different treatment histories, need a longer duration of treatment, compared to those who are being referred having already completed other treatments. This will help determine whether the current findings are generalisable to adolescents, or specific to the current group characteristics. Research is also needed to determine whether transdiagnostic treatments that target social and general functioning have added benefits for adolescents who struggle with diagnostic labels and for whom these may have a negative impact in terms of developing self-identity.

## Conclusion

This is the first study to report on the adolescent experience of a slightly age modified RO DBT. Findings suggest it is useful in both content and process, although materials may need to be adapted/abbreviated further for the adolescent group.

## Supplementary Information

**Additional file 1.** RO DBT: Interview topic guide

## Data Availability

Data generated, analysed and reported here are not publicly available due to it being potentially identifying, but are available in a slightly shortened, de-identified form from the corresponding author on reasonable request.

## Abbreviations

%mBMI: Percentage of median body mass index
ASC-WP: Assessing Styles of Coping Word-pair Checklist
CAMHS: Child and Adolescent Mental Health Services
CBT: Cognitive Behavioural Therapy
DAWBA: Development and Wellbeing Assessment
DBT: Dialectical Behaviour Therapy
DSM-5: Diagnostic and Statistical Manual, 5th edition
EDE: Eating Disorder Examination
FT-ED: Eating disorder focused family therapy
ICD-10: International Classification of Diseases, 10th edition
MCCAED: Maudsley Centre for Child and Adolescent Eating Disorders
OCD: Obsessive Compulsive Disorder
RO-A: Adolescent adaptation of Radically Open Dialectical Behaviour Therapy
RO DBT: Radically Open Dialectical Behaviour Therapy
SLaM: South London and Maudsley

## References

[1] Lynch TR (2018). Radically open dialectical behavior therapy: theory and practice for treating disorders of overcontrol.

[2] Lynch TR (2018). The skills training manual for radically open dialectical behavior therapy: a Clinician’s guide for treating disorders of Overcontrol.

[3] Gilbert K, Perino MT, Myers MJ, Sylvester CM (2020). Overcontrol and neural response to errors in pediatric anxiety disorders. J Anxiety Disord.

[4] Hempel R, Vanderbleek E, Lynch TR (2018). Radically open DBT: targeting emotional loneliness in anorexia nervosa. Eat Disord.

[5] Lynch TR, Hempel RJ, Whalley B, Byford S, Chamba R, Clarke P (2020). Refractory depression – mechanisms and efficacy of radically open dialectical behaviour therapy (RefraMED): findings of a randomised trial on benefits and harms. Br J Psychiatry.

[6] Kaye WH, Wierenga CE, Bailer UF, Simmons AN, Bischoff-Grethe A (2013). Nothing tastes as good as skinny feels: the neurobiology of anorexia nervosa. Trends Neurosci.

[7] Baudinet J, Simic M, Griffiths H, Donnelly C, Stewart C, Goddard E (2020). Targeting maladaptive overcontrol with radically open dialectical behaviour therapy in a day programme for adolescents with restrictive eating disorders: an uncontrolled case series. J Eat Disord.

[8] Blinder BJ, Cumella EJ, Sanathara VA. Psychiatric comorbidities of female inpatients with eating disorders. Psychosom Med. 2006;68(3):454-2. 10.1097/01.psy.0000221254.77675.f5 16738079

[9] Kaye WH, Bulik CM, Thornton L, Barbarich N, Masters K (2004). Comorbidity of anxiety disorders with anorexia and bulimia nervosa. Am J Psychiatry..

[10] Smith R, Shepard C, Wiltgen A, Rufino K, Fowler JC (2017). Treatment outcomes for inpatients with obsessive-compulsive personality disorder: an open comparison trial. J Affect Disord.

[11] Khalsa SS, Portnoff LC, McCurdy-McKinnon D, Feusner JD (2017). What happens after treatment? A systematic review of relapse, remission, and recovery in anorexia nervosa. J Eat Disord.

[12] Negt P, Brakemeier EL, Michalak J, Winter L, Bleich S, Kahl KG (2016). The treatment of chronic depression with cognitive behavioral analysis system of psychotherapy: a systematic review and meta-analysis of randomized-controlled clinical trials. Brain Behav.

[13] Richard M, Bauer S, Kordy H, COST Action B6 (2005). Relapse in anorexia and bulimia nervosa—a 2.5-year follow-up study. Eur Eat Disorders Rev.

[14] Keel PK, Dorer DJ, Franko DL, Jackson SC, Herzog DB (2005). Postremission predictors of relapse in women with eating disorders. Am J Psychiatry.

[15] Fournier JC, DeRubeis RJ, Shelton RC, Hollon SD, Amsterdam JD, Gallop R (2009). Prediction of response to medication and cognitive therapy in the treatment of moderate to severe depression. J Consult Clin Psychol.

[16] Berkman ND, Lohr KN, Bulik CM (2007). Outcomes of eating disorders: a systematic review of the literature. Int J Eat Disord.

[17] Isaksson M, Ghaderi A, Ramklint M, Wolf-Arehult M (2021). Radically open dialectical behavior therapy for anorexia nervosa: a multiple baseline single-case experimental design study across 13 cases. J Behav Ther Exp Psychiatry.

[18] Lynch TR, Gray KL, Hempel RJ, Titley M, Chen EY, O’Mahen HA (2013). Radically open-dialectical behavior therapy for adult anorexia nervosa: feasibility and outcomes from an inpatient program. BMC Psychiatry.

[19] Cornwall P, Simpson S, Gibbs C, Morfee V. Evaluation of radically open dialectical behaviour therapy in an adult community mental health team: effectiveness in people with autism spectrum disorders. BJPsych Bull. 2021;45(3):146-53. 10.1192/bjb.2020.113. 10.1192/bjb.2020.113 PMC9059310 33261708

[20] Isaksson M, Ghaderi A, Wolf-Arehult M, Öster C, Ramklint M (2021). Sharing and connecting with others – patient experiences of radically open dialectical behavior therapy for anorexia nervosa and overcontrol: a qualitative study. J Eat Disord.

[21] Baudinet J, Stewart C, Bennett E, Konstantellou A, Parham R, Smith K (2021). Radically open dialectical behaviour therapy adapted for adolescents: a case series. BMC Psychiatry..

[22] Goodman R, Ford T, Richards H, Gatward R, Meltzer H (2000). The development and well-being assessment: description and initial validation of an integrated assessment of child and adolescent psychopathology. J Child Psychol Psychiatry.

[23] World Health Organization (2004). International statistical classification of diseases and related health problems (ICD-10). 10th revision.

[24] American Psychiatric Association (2013). Diagnostic and statistical manual of mental disorders: DSM-5.

[25] Aebi M, Kuhn C, Metzke CW, Stringaris A, Goodman R, Steinhausen HC. The use of the development and well-being assessment (DAWBA) in clinical practice: a randomized trial. Eur Child Adolesc Psychiatry. 2012;21(10):559-67. 10.1007/s00787-012-0293-6. 10.1007/s00787-012-0293-6 PMC3866649 22722664

[26] Lenz, AS, James, P, Stewart, C et al. A Preliminary Validation of the Youth Over- and Under-Control (YOU-C) Screening Measure with a Community Sample. Int J Adv Counselling. 2021;43:489–503. 10.1007/s10447-021-09439-9.

[27] Braun V, Clarke V. One size fits all? What counts as quality practice in (reflexive) thematic analysis? Qual Res Psychol. 2020:1–25.

[28] Braun V, Clarke V (2013). Successful qualitative research: a practical guide for beginners.

[29] Braun V, Clarke V (2006). Using thematic analysis in psychology. Qual Res Psychol.

[30] de Haan AM, Boon AE, de Jong JTVM, Hoeve M, Vermeiren RRJM (2013). A meta-analytic review on treatment dropout in child and adolescent outpatient mental health care. Clin Psychol Rev.

