# Supplementary material for: Adolescent experience of radically open dialectical behaviour therapy: a qualitative study
Source: BMC Psychiatry. 2022 Jul 14;22:466. doi: 10.1186/s12888-022-04114-8 (PMC9281135; doi:10.1186/s12888-022-04114-8)
Supplement: Supplementary file 1 — Additional file 1. RO DBT: Interview topic guide [file 12888_2022_4114_MOESM1_ESM.pdf]

RO DBT: INTERVIEW TOPIC GUIDE

- How would you describe RO DBT to a friend?

- How did RO DBT differ from treatment that you have had before?

- Did RO DBT seem relevant to your experiences and difficulties (i.e. did it make sense why it was offered to you?).

  Follow up: in what ways?

- What has stuck with you from the programme? What do you use? What ideas/ways of thinking about things have been interesting to you?

- Are there things about the programme that felt less relevant?

  Follow-up: what were they /could you say a bit more

- Have there been elements of the programme that you have found challenging?

  Follow up: what were these? Looking back do you think the challenges were helpful…?

- Has RO DBT made a difference to the ways that you manage things now?

  Follow up: in terms of how you manage your social relationships or interact with people, or manage situations that you find difficult?

  Follow up: in what ways?

- Is there anything that stands out as helpful/unhelpful from the skills class (group)/individual sessions?

- Any final thoughts/Anything else you want to add?
